# Relationships between respiratory parameters and quadriceps strength in subjects with chronic obstructive pulmonary disease

**DOI:** 10.1080/07853890.2021.1896688

**Published:** 2021-09-28

**Authors:** Gabriel Mahassine, Tiago Vargas, Renato Rito, Diogo Lopes, Ângela Maria Pereira

**Affiliations:** a Department of Physiotherapy, Escola Superior de Saúde Egas Moniz (ESSEM), Egas Moniz Cooperativa de Ensino Superior, Caparica, Portugal; b Centro de Investigação Interdisciplinar Egas Moniz (CiiEM), Egas Moniz Cooperativa de Ensino Superior, Caparica, Portugal; c Hospital Garcia de Orta, Almada, Portugal

## Abstract

**Introduction:**

Exercise capacity in chronic obstructive pulmonary disease (COPD) patients depends on the degree of airflow obstruction, the severity of the hypoxaemia and skeletal muscle function. Muscle atrophy and weakness are considered systemic consequences of COPD and are associated with reduced exercise capacity [1]. Peripheral muscle weakness is a systemic manifestation of COPD which influences exercise limitation, quality of life and prognosis in most of the patients. Chronic hypoxaemia resulting from COPD may increase the pathophysiological mechanisms involved in peripheral muscle dysfunction namely chronic inflammation and oxidative stress, deconditioning leading to muscle mass loss [2]. The purpose of this study is to asses respiratory parameters, maximum voluntary contraction quadriceps muscle and their relations in COPD subjects.

**Materials and methods:**

An observational study was performed with inclusion of thirty men with moderate COPD, FEV1, 46.5 ± 12.6%, 64.4 ± 6.3 years old; weight, 76.4 ± 12.8 kg; height, 170.9 ± 4.9 cm, effort subjective perception (ESP) 17.1 ± 1.5, and dyspnoea subjective perception (DSP) 5.27 ± 2.4. Spirometry and 1-RM were used as evaluation methods. Before initiation all subjects performed spirometry (DATOSPIR-120 Sibelmed, Spain) according to American Thoracic Society (ATS) guidelines and FEV1 was measured. The maximum voluntary contraction was assessed by the one repetition maximum (1-RM) strength test, which was performed using a resistance weight-lifting machine (Leg Extension, Salter®, Commercial Salter, S.A. Spain). The study was approved by the Ethics Committee of the Garcia de Orta Hospital and all participants gave their informed consent

**Results:**

Our results showed that as FEV1 and ESP increases, the quadriceps muscle strength also increase, with Pearson correlation values of *r* = 0.585 (*p* < .01) and *r* = 0.577 (*p* < .01), respectively. On the other hand, 1-RM was also influenced by DSP with Pearson coefficient *r*=–0.413 (*p* < .01). In relation to FEV1, as it increases, ESP and DSP tend to decrease their values, as evidenced by the coefficient of Pearson values obtained *r*=–0.623 (*p* < .01) and *r*=–0.670 (*p* < .01), respectively.

**Discussion and conclusions:**

In this study, we can verify that quadriceps strength is related to the severity of airflow obstruction as measured by FEV1. We found significant correlations between quadriceps strength and FEV1. These findings are consistent with some previous studies finding a significant association between exercise capacity and lung function [3]. So, we can also conclude that as quadriceps strength increase, dyspnoea and effort subjective perceptions decrease, highlighting the need to include quadriceps training in COPD rehabilitation programmes.
